# N-(4-Hydroxyphenyl) Retinamide Potentiated Anti-Tumor Efficacy of Genistein in Human Ewing’s Sarcoma Xenografts

**DOI:** 10.4021/wjon301w

**Published:** 2011-04-09

**Authors:** Surajit Karmakar, Subhasree Roy Choudhury, Naren L. Banik, Swapan K. Ray

**Affiliations:** a Department of Pathology, Microbiology, and Immunology, University of South Carolina School of Medicine, Columbia, SC 29209, USA; b Department of Neurosciences, Medical University of South Carolina, Charleston, SC 29425, USA

**Keywords:** Apoptosis, Caspases, Ewing’s sarcoma, Genistein, N-(4-hydroxyphenyl) retinamide

## Abstract

**Background:**

Ewing’s sarcoma is a pediatric tumor that mainly occurs in soft tissues and bones. New therapeutic strategies are urgently needed for treatment of Ewing’s sarcoma. We examined for the first time the efficacy of N-(4-hydroxyphenyl) retinamide (4-HPR) and genistein (GST) alone and also in combination for controlling growth of human Ewing’s sarcoma SK-N-MC and RD-ES xenografts.

**Methods:**

Efficacy of combination therapy was evaluated using histopathological parameters. Molecular mechanisms of combination therapy were detected using Western blotting and immunofluorescence microscopy.

**Results:**

Histopathological examination of tumor sections showed that control group maintained characteristic growth of tumors, 4-HPR alone inhibited proliferation of tumor cells, GST alone induced apoptosis to some extent, and combination of 4-HPR and GST significantly induced apoptosis in both Ewing’s sarcoma xenografts. Time-dependent reductions in body weight, tumor volume, and tumor weight were also found. Combination therapy increased Bax : Bcl-2 ratio to trigger mitochondrial release of Smac/Diablo into the cytosol to downregulate the baculovirus inhibitor-of-apoptosis repeat containing (BIRC) proteins such as BIRC-2 and BIRC-3 and thereby promote apoptosis. Activation of caspase-3 and mitochondrial release of apoptosis-inducing factor (AIF) occurred in course of apoptosis. Downregulation of the survival factor NF-κB and the angiogenic factors VEGF and FGF2 and increase in caspase-3 activity controlled tumor growth. *In situ* immunofluorescent labelings showed overexpression of calpain, caspase-12 and caspase-3, and AIF in xenografts, indicating induction of cysteine proteases and AIF for apoptosis.

**Conclusions:**

Results revealed that combination of 4-HPR and GST could be highly effective treatment for inhibiting Ewing’s sarcomas *in vivo*.

## Introduction

Ewing’s sarcoma family of tumors (ESFT) includes Ewing’s sarcoma, Askin’s tumor and primitive neuroectodermal tumors (PNET) that commonly occur in soft tissues and bone in children and young adults [1]. Ewing’s sarcoma is the second most common malignant bone tumor that accounts for 10 - 15% of all primary bone tumors in pediatric and adolescence cancers [1, 2]. Ewing’s sarcoma is characterized by poor neural differentiation, chromosomal translocation at t(11;22)(q24;q12) with high expression of the oncogenic chimeric transcription factor EWS-FLI1 fusion protein [3]. Current therapeutic strategies include local surgery and radiotherapy in conjunction with systemic chemotherapy followed by stem cell transplantation [4]. However, therapeutic inefficacy in most cases accounts for approximately less than 20% long-term survival of metastatic Ewing’s sarcoma patients [5, 6]. So, there is an urgent need for development of new and effective therapeutic strategies for treating these childhood tumors.

The versatile biological effects of retinoids include promotion of differentiation, inhibition of proliferation, and induction of apoptosis in different cancers [7]. The differentiating ability and anti-cancer property of the retinoids such as all-*trans* retinoic acid (ATRA) and 13-*cis* retinoic acid (13-CRA) are well established in several experimental *in vitro* and *in vivo* models [8, 9]. Fenretinide or N-(4-Hydroxyphenyl) retinamide (4-HPR) is a promising anti-tumor agent among the synthetic and natural retinoids including ATRA and 13-CRA [10]. 4-HPR shows broad spectrum anti-cancer properties in a variety of *in vitro* and *in vivo* studies [11]. Most of the *in vitro* studies using 4-HPR reported its anti-tumor activity due to induction of apoptosis [11] with increased Bax : Bcl-2 ratio and caspase-3 activation in glioblastoma [12] and also Ewing’s sarcoma [13] cells. The effects of 4-HPR are dose-dependent and it at relatively low concentrations induces neuronal differentiation in human neuroblastoma SH-SY5Y cells [14] and retinal pigment epithelial (ARPE-19) cells due to differential expression of a variety of key proteins [15]. The differentiating and growth inhibitory properties of 4-HPR indicate that it can be therapeutically useful in combination with another cytotoxic agent against Ewing’s sarcoma.

Genistein (GST), a soy-derived isoflavonoid inhibits the growth of various cancer cells through modulation of genes that control cell cycle and apoptosis [16]. GST induced apoptosis with alterations of Bax and Bcl-2 levels and increase in caspase-3 activity in human breast cancer MDA-MB-231 cells [17]. GST induced endoplasmic reticulum (ER) stress and mitochondrial damage in human hepatoma Hep3B cells [18] and Ca^2+^-mediated calpain/caspase-12-dependent apoptosis in breast cancer MCF-7 cells [19]. Previously, we reported that GST induced activation of calpain and caspases for apoptosis in human neuroblastoma SH-SY5Y cells [20]. Both 4-HPR [21, 22] and GST [23, 24] showed anti-proliferative and anti-angiogenic properties due to inhibition of NF-κB, VEGF, and FGF2 in a variety of cancer cell lines.

In this investigation, we explored the efficacy of 4-HPR and GST alone and in combination for induction of apoptosis in two human Ewing’s sarcoma SK-N-MC and RD-ES xenografts in nude mice. There has not yet been any report on this combination therapy in Ewing’s sarcoma. Some sparse reports demonstrated that 4-HPR pre-treatment with cisplatin, etoposide, or carboplatin in neuroblastoma cell lines [25] and 4-HPR combination treatment with celecoxib in lung cancer NSCLC cells [26] synergistically increased more apoptosis than single treatment. On the other hand, GST in combination with Ara-C in acute myeloid leukemia [27] and with SB715992 in prostate cancer PC-3 cells [28] synergistically increased more therapeutic outcome than single treatment. GST and gemcitabine combination is much more effective than either agent alone in *in vitro* and *in vivo* pancreatic cancer models [29]. GST potentiated cisplatin-induced anti-tumor activity in pancreatic tumor BxPC-3 xenografts [30]. In this investigation, our results showed that combination of 4-HPR and GST produced better efficacy than single treatment for activation of multiple molecular mechanisms for apoptosis in human Ewing’s sarcoma SK-N-MC and RD-ES xenografts in nude mice.

## Materials and Methods

### Tumor cell lines and culture conditions

Human Ewing’s sarcoma SK-N-MC and RD-ES cell lines were purchased from the American Type Culture Collection (ATCC, Manassas, VA, USA). Cells were grown in 75-cm^2^ flasks containing 10 ml of 1 x RPMI 1640 supplemented with 10% fetal bovine serum (FBS) and 1% penicillin and streptomycin in a fully-humidified incubator containing 5% CO_2_ at 37 °C. 4-HPR and GST were purchased from Sigma (St. Louis, MO, USA). Dimethyl sulfoxide (DMSO) was used as vehicle to make stock solutions of 4-HPR and GST and all aliquots of stock solutions were stored at -20 °C until ready to use. Aliquots were serially diluted so as to achieve the desired final concentrations of drugs. For animal treatments, drugs were appropriately diluted in 0.9% saline before treatments. Final concentration of DMSO (< 0.01%) did not affect the growth of xenografts.

### Ewing’s sarcoma xenografts in nude mice

Six weeks-old female athymic nu/nu mice were obtained from Charles River Laboratories (Wilmington, MA, USA). All animal studies were conducted according to the NIH guidelines and also approved by our Institutional Animal Care and Use Committee (IACUC). Each of Ewing’s sarcoma SK-N-MC and RD-ES cells (6 x 10^6^) in 100 µl of 1 : 1 mixture with Matrigel (BD Biosciences, San Jose, CA, USA) was implanted by subcutaneous (sc) injection into the flank of each mouse under isoflurane anesthesia condition. Palpable xenografts developed within 6 to 8 days, tumors were measured using an external caliper, and tumor volume was calculated using the formula: 4π/3 x (length/2) x (width/2)^2^. Animals with 3 weeks-old Ewing’s sarcoma xenografts were randomly divided into 4 groups: control (CTL), 4-HPR, GST, and 4-HPR plus GST. Animals in CTL group did not receive any therapy. Each animal in other groups received intraperitoneally (ip) a daily dose of 4-HPR (20 µg/kg/day), GST (2 mg/kg/day), or 4-HPR (20 µg/kg/day) + 4-h later GST (2 mg/kg/day) for 8 or 15 days. After treatments for 8 or 15 days, we determined tumor volume and weight. For time-course studies, once in every week we monitored animal body weight and tumor volume for 21 days before the treatments and during the treatments for the next 15 days.

### Histopathological examination of xenograft sections

After completion of treatment schedule, xenografts were excised. One half of each tumor was immediately frozen in liquid nitrogen and stored at -80 °C. The other half of tumor was immediately frozen (-80 °C) in Optima Cutting Temperature media (Fisher Scientific, Suwanee, GA, USA) and 5 µm sections were cut with a cryostat. These sections were subjected to hematoxylin and eosin (H&E) staining for examination of changes in histopathology following treatments, as we reported previously [31].

### *In situ* immunofluorescent labeling for detecting expression of pro-apoptotic protein

Tumor sections were blocked with 2% (v/v) horse and goat sera (Sigma, St. Louis, MO, USA) in PBS for 1 h and then probed with a primary IgG antibody (1 : 100) for 1 h and rinsed in PBS. Slides were then incubated with either FITC-conjugated or Texas red-conjugated secondary IgG antibody (1 : 75) (Vector Laboratories, Burlingame, CA, USA) for 1 h and rinsed in PBS and water. Then, slides were mounted with the anti-fade medium Vectashield (Vector Laboratories) and viewed promptly under a fluorescence microscope at 200 x magnification (Olympus, Japan). The digital pictures were captured using Image-Pro Plus software (Media Cybernetics, Silver Spring, MD, USA), as we described previously [32].

### Combined TUNEL and double immunofluorescent staining for detection of apoptosis and upregulation of pro-apoptotic protein

We pre-fixed 5 µm sections in 95% ethanol (10 min) and 4% methanol-free formaldehyde (10 min) (Polysciences, Warrington, PA, USA) and washed in PBS. After equilibration in terminal deoxynucleotidyl transferase (TdT) buffer (Promega, Madison, WI, USA), sections were incubated with digoxigenin (DIG)-labeled nucleotides (Roche, Indianapolis, IN, USA) and recombinant TdT (Promega) at 37 °C for 1 h. TUNEL reaction was stopped and unbound nucleotides were removed by washing in PBS. Slides were blocked with 2% normal goat and horse sera, incubated with a primary IgG antibody (1 : 100) for 1 h, washed in PBS, and incubated with flouresceinated antibodies such as Texas red-conjugated anti-DIG antibody (1 : 50) (Roche, Indianapolis, IN, USA) and FITC-conjugated secondary antibody (1 : 75) (Vector Laboratories) for 30 min [33]. Slides were then washed in PBS and water, mounted with VectaShield (Vector Laboratories), and examined under a fluorescence microscope (Olympus) at 200 x magnification to capture images using Image Pro Plus Software (Media Cybernetics).

### Western blotting

We reported this method previously [32, 34] and modified it to use in this investigation. Briefly, protein samples were extracted following the lysis of control and drug-treated tumor tissues, quantitated spectrophotometrically, denatured in boiling water for 5 min, and loaded onto the SDS-polyacrylamide gradient (4 - 20% or 5%) gels (Bio-Rad, Hercules, CA, USA). All proteins were resolved by electrophoresis and then electroblotted to the membranes. The blots were incubated with a primary IgG antibody followed by incubation with an alkaline horseradish peroxidase (HRP)-conjuaged secondary IgG antibody. Subsequently, specific protein bands were detected by HRP/H_2_O_2_-catalyzed oxidation of luminol in alkaline conditions using the enhanced chemiluminescence system and autoradiography.

### Colorimetric assay for determination of caspase-3 activity

Caspase-3 activity in the lysates of xenografts was measured using the commercially available caspase-3 assay kit (Sigma, St. Louis, MO, USA).

### Statistical analysis

Western blots and immunofluorescent images were used to quantify total number of pixels above the background by using the public domain NIH Image 1.63 software. Data were analyzed using Minitab® 15 Statistical Software (Minitab, State College, PA, USA), expressed as arbitrary units ± standard error of mean (SEM) of separate experiments (n ≥ 3), and compared by one-way analysis of variance (ANOVA) followed by Fisher post-hoc test. Significant difference between control and a treatment was indicated by *P < 0.05 or **P < 0.001.

## Results

### Tumor volume, weight, and histopathological evaluations

**Figure 1:**
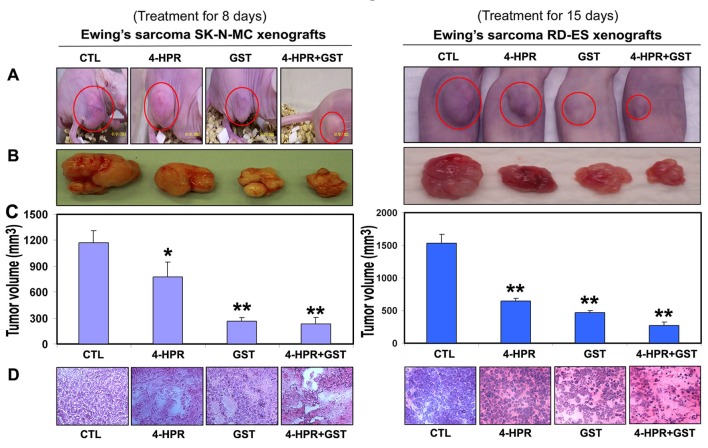
Regression of Ewing’s sarcoma and histopathological changes in xenografts. (A) Mice with SK-N-MC and RD-ES xenografts; (B) representative tumors; (C) tumor volume; and (D) histopathological changes after the treatments. Mice with xenografts were treated for 8 or 15 days. Treatments: CTL, 4-HPR (20 µg/kg/day), GST (2 mg/kg/day), and 4-HPR (20 µg/kg/day) plus 4-h later GST (2 mg/kg/day). We used 6 animals per group (*P < 0.05 or **P < 0.001).

**Figure 2:**
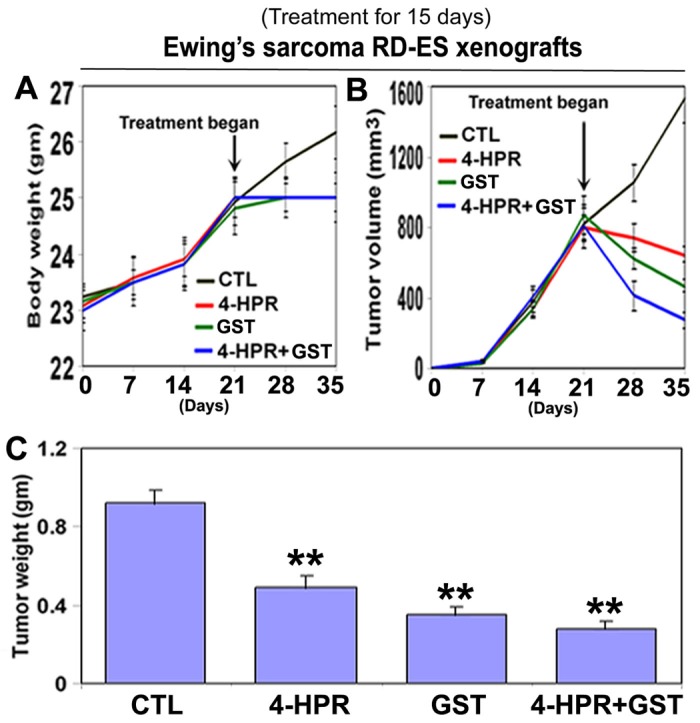
Time-dependent reduction in animal body weight, tumor volume, and tumor weight in RD-ES xenografts following treatments. Treatments for 15 days: control (CTL), 4-HPR (20 µg/kg/day), GST (2 mg/kg/day), and 4-HPR (20 µg/kg/day) plus 4-h later GST (2 mg/kg/day). (A) Body weight changes; (B) tumor volume changes; and (C) tumor weight changes after therapies. Combination therapy showed best efficacy in reducing animal body weight and tumor volume. We used 6 animals per group (*P < 0.05 or **P < 0.001).

We used two strategies in Ewing’s sarcoma xenografts to determine the efficacy of treatments (Fig. 1). In the first strategy (left panels), we treated SK-N-MC xenografts in nude mice for 8 days; and in the second strategy (right panels), we treated RD-ES xenografts in nude mice for 15 days (Fig. 1A). Compared with CTL or a monotherapy, 4-HPR plus GST showed significant reductions in tumor volume, and combination therapy for 15 days showed more tumor regression than combination therapy for 8 days (Fig. 1B, C). Following treatments for 8 or 15 days, H&E staining of tumor sections showed that CTL tumors maintained characteristic growth, 4-HPR alone inhibited tumor cell proliferation, GST alone induced cell death to some extent, and 4-HPR plus GST increased cell death; and extent of cell death was more due to treatment with 4-HPR plus GST for 15 days than for 8 days (Fig. 1D). Time-dependently, 4-HPR plus GST caused reductions in animal body weight (Fig. 2A), tumor volume (Fig. 2B), and tumor weight (Fig. 2C) in Ewing’s sarcoma RD-ES xenografts, compared with corresponding CTL groups.

### Combination therapy increased Bax : Bcl-2 ratio

**Figure 3:**
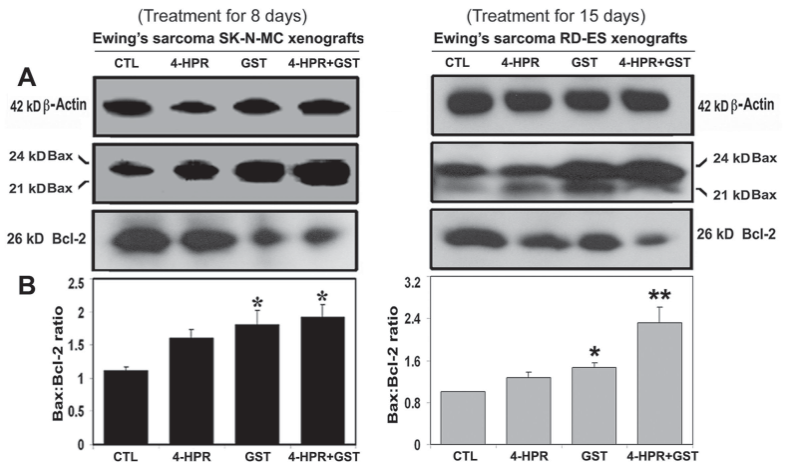
Western blotting for determination of Bax : Bcl-2 ratio in Ewing’s sarcoma SK-N-MC and RD-ES xenografts. Treatments for 8 or 15 days: CTL, 4-HPR (20 µg/kg/day), GST (2 mg/kg/day), and 4-HPR (20 µg/kg/day) plus 4-h later GST (2 mg/kg/day). (A) Representative Western blots (n ≥ 3) showed expression of 42 kD β-actin, 21 and 24 kD Bax, and 26 kD Bcl-2, and (B) changes in Bax : Bcl-2 ratio in SK-N-MC and RD-ES xenografts. Significant difference between CTL and a treatment was indicated by *P < 0.05 or **P < 0.001.

Western blotting showed alterations in the expression of Bax and Bcl-2 proteins in Ewing’s sarcoma SK-N-MC and RD-ES xenografts (Fig. 3). Expression of 42 kD β-actin was used as a loading control (Fig. 3A). Treatment with 4-HPR plus GST increased the Bax : Bcl-2 ratio in both xenografts (Fig. 3B). Increase in Bax : Bcl-2 ratio could cause mitochondrial release of pro-apoptotic molecules to trigger activation of downstream processes of apoptosis.

### Mitochondrial release of pro-apoptotic molecules, activation of caspase-3, and downregulation of survival and angiogenetic factors

**Figure 4:**
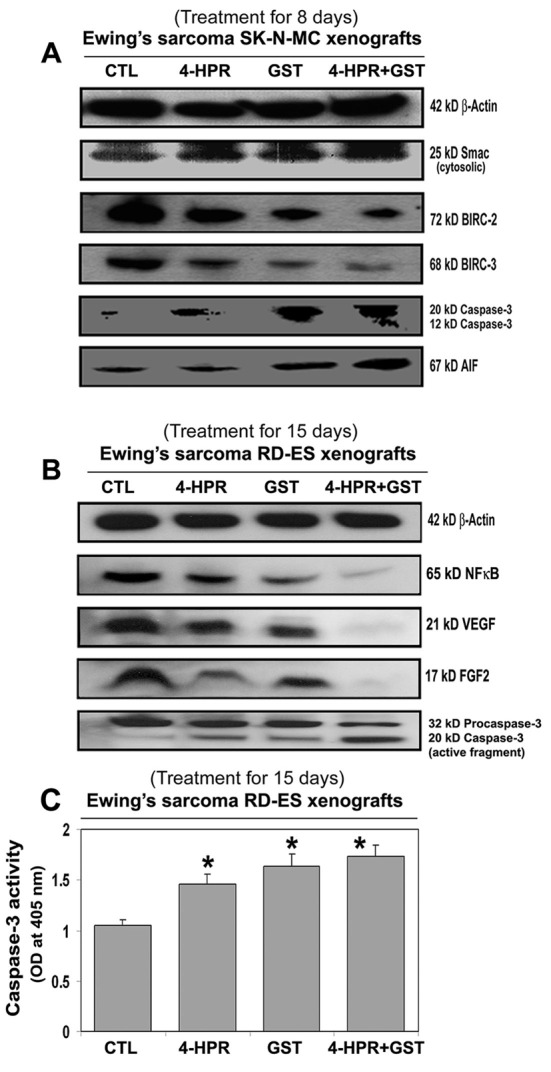
Changes in expression of pro-apoptotic and anti-apoptotic proteins in Ewing’s sarcoma xenografts. Treatments for 8 or 15 days: CTL, 4-HPR (20 µg/kg/day), GST (2 mg/kg/day), and 4-HPR (20 µg/kg/day) plus 4-h later GST (2 mg/kg/day). Representative Western blots (n ≥ 3) showed expression of (A) 42 kD β-actin, 25 kD Smac/Diablo, 72 kD BIRC-2, 68 kD BIRC-3, 20 and 12 kD active caspase-3, and 67 kD AIF in SK-N-MC xenografts; and (B) 42 kD β-actin, 65 kD NF-κB, 21 kD VEGF, 17 kD FGF2, and 32 and 20 kD caspase-3 in RD-ES xenografts. (C) Colorimetric assay for determination of caspase-3 activity in RD-ES xenografts. Significant difference between CTL and a treatment was indicated by *P < 0.05 or **P < 0.001.

Western blotting (Fig. 4) showed the most increases in mitochondrial release of 25 kD Smac into the cytosol and downregulation of 72 kD BIRC-2 and 68 kD BIRC-3 to favor activation of caspase-3 (Fig. 4A) for apoptosis following combination therapy in SK-N-MC xenografts. An increase in cytosolic level of 67 kD AIF after treatment with 4-HPR plus GST (Fig. 4A) indicated activation of caspase-independent pathway of apoptosis as well.

Also, 4-HPR plus GST caused the highest downregulation of the cell survival factor 65 kD NF-κB and the angiogenetic factors such as 21 kD VEGF and 17 kD FGF2 and also activation of caspase-3 for apoptosis in RD-ES xenografts (Fig. 4B). Further, an increase in caspase-3 activity in course of apoptosis in Ewing’s sarcoma RD-ES xenografts was measured by a colorimetric assay (Fig. 4C). The release of free *p*-nitroaniline (*p*NA) moiety (yellow product) due to hydrolysis of the specific substrate Ac-DEVD-*p*NA by caspase-3 activity in xenografts most significantly occurred following treatment with 4-HPR plus GST, compared with CTL or monotherapy groups (Fig. 4C).

### Involvement of calpain and caspase-12 in apoptosis in xenografts

**Figure 5:**
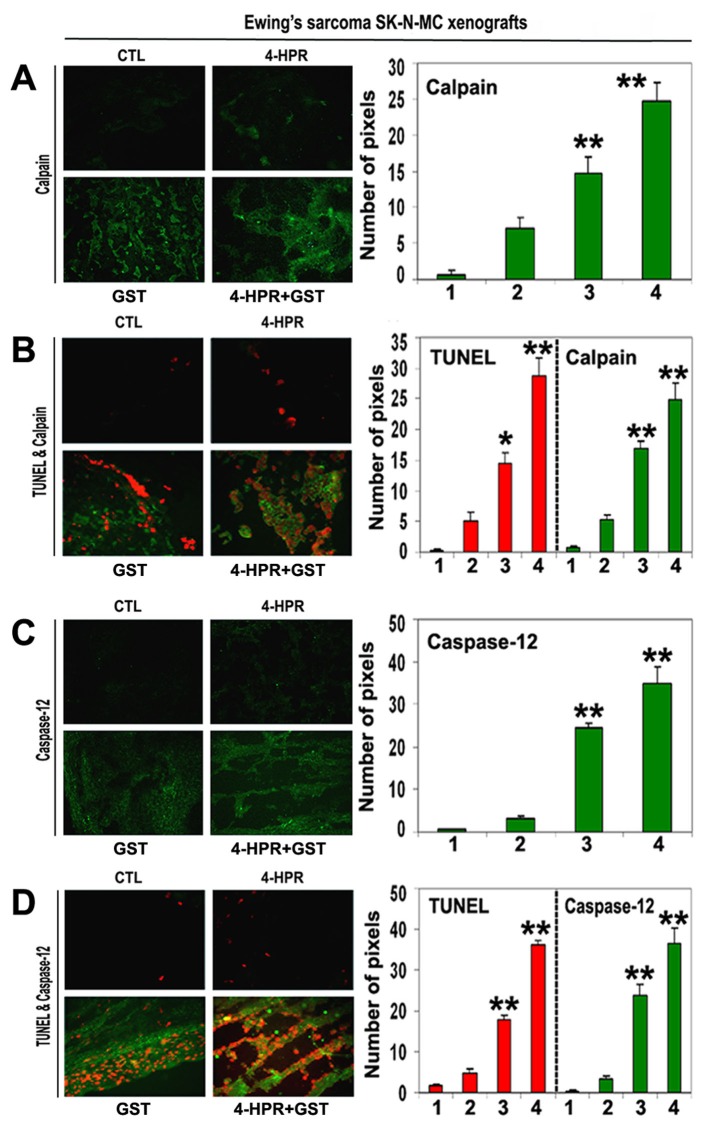
In situ single and double immunofluorescent labelings to detect increases in expression of calpain and caspase-12 during apoptosis (TUNEL-positive cells) in SK-N-MC xenografts. Treatments for 8 days: CTL, 4-HPR (20 µg/kg/day), GST (2 mg/kg/day), and 4-HPR (20 µg/kg/day) plus 4-h later GST (2 mg/kg/day). (A) Combination therapy most significantly increased calpain expression in SK-N-MC xenografts. (B) Calpain expression in TUNEL-positive cells. (C) Combination therapy most significantly increased caspase-12 expression in SK-N-MC xenografts. (D) Caspase-12 expression in TUNEL-positive cells. In bar graphs: 1 = CTL, 2 = 4-HPR, 3 = GST, and 4 = 4-HPR plus GST. Significant difference between CTL and a treatment was indicated by *P < 0.05 or **P < 0.001.

We employed single immunofluorescent (SIF) staining to detect expression of specific pro-apoptotic protein (Fig. 5) whereas double immunofluorescent (DIF) staining to detect expression of specific pro-apoptotic protein and DNA fragmentation in apoptotic cells in the xenograft sections (Fig. 5). SIF staining showed a significant increase in calpain (Fig. 5A) and DIF staining detected significant overexpression of calpain and increase in DNA fragmentation in Ewing’s sarcoma RD-ES xenografts following treatment with 4-HPR plus GST (Fig. 5B), indicating calpain upregulation for apoptosis in Ewing’s sarcoma SK-N-MC xenografts.

Also, SIF staining detected significant increase in expression of caspase-12 following combination therapy (Fig. 5C). DIF staining showed a prominent role for caspase-12 in cell death (Fig. 5D). We identified overexpression of caspase-12 and increase in DNA fragmentation in Ewing’s sarcoma SK-N-MC xenografts following treatment with 4-HPR plus GST (Fig. 5D).

### Activation of caspase-dependent and caspase-independent pathways for apoptosis

**Figure 6:**
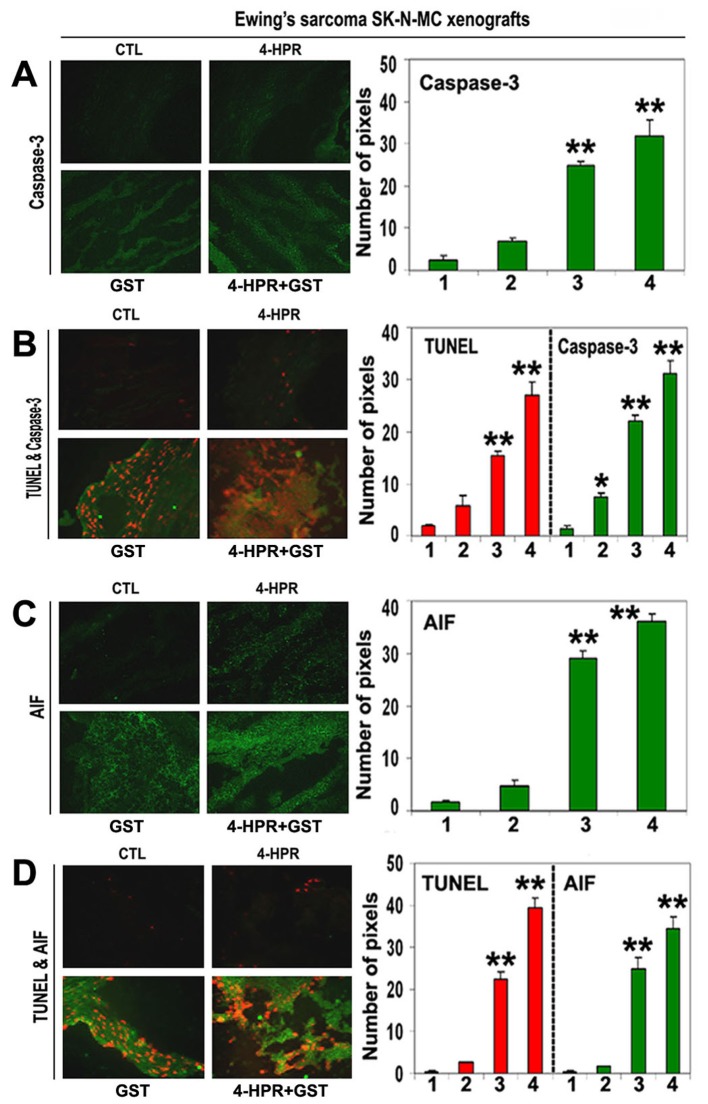
In situ single and double immunofluorescent labelings to detect increases in expression of caspase-3 and AIF during apoptosis (TUNEL-positive cells) in SK-N-MC xenografts. Treatments for 8 days: CTL, 4-HPR (20 µg/kg/day), GST (2 mg/kg/day), and 4-HPR (20 µg/kg/day) plus 4-h later GST (2 mg/kg/day). (A) Combination therapy most significantly increased caspase-3 expression in SK-N-MC xenografts. (B) Caspase-3 expression in TUNEL-positive cells. (C) Combination therapy most significantly increased AIF expression in SK-N-MC xenografts. (D) AIF expression in TUNEL-positive cells. In bar graphs: 1 = CTL, 2 = 4-HPR, 3 = GST, and 4 = 4-HPR plus GST. Significant difference between CTL and a treatment was indicated by *P < 0.05 or **P < 0.001.

We used SIF staining to examine expression of caspase-3 and found significant overexpression of caspase-3 in the Ewing’s sarcoma SK-N-MC xenografts after treatment with 4-HPR plus GST (Fig. 6A). DIF staining showed significant increases in expression of caspase-3 and DNA fragmentation after the combination therapy, indicating an essential role for caspase-3 in apoptotic DNA fragmentation in the Ewing’s sarcoma SK-N-MC xenografts (Fig. 6B).

To identify caspase-independent mechanism in apoptosis, we examined the expression of AIF using SIF staining and found significant increase in expression of AIF in the SK-N-MC xenografts after treatment with 4-HPR plus GST (Fig. 6C). Overexpression of AIF and increase in DNA fragmentation occurred most significantly in the Ewing’s sarcoma xenografts after combination therapy (Fig. 6D). These observations showed the highest induction of caspase-dependent pathway as well as caspase-independent pathway for apoptosis in Ewing’s sarcoma SK-N-MC xenografts after treatment with 4-HPR plus GST.

## Discussion

In this investigation, we explored the efficacy of combination of 4-HPR and GST for controlling the growth of human Ewing’s sarcoma xenografts in nude mice. Evidently, combination of 4-HPR and GST produced anti-tumor effects in two *in vivo* pre-clinical models of Ewing's sarcoma with activation of multiple molecular mechanisms for apoptosis (Fig. 1-6).

We found that combination of 4-HPR and GST significantly reduced the tumor volume in both Ewing’s sarcoma xenografts due to prevention cell proliferation and induction of cell death (Fig. 1 and 2). Previous studies reported that 4-HPR or GST induced apoptosis in a variety of cell lines [11, 15, 20, 35] including Ewing’s sarcoma [13, 36] with activation of caspase-3. But this is the first report that treatment with combination of 4-HPR and GST shows better efficacy than monotherapy in two Ewing’s sarcoma xenografts. An increase in Bax : Bcl-2 ratio is a key determining factor for induction of apoptosis [37, 38]. In both Ewing’s sarcoma SK-N-MC and RD-ES xenografts, there were significant increases in Bax : Bcl-2 ratio (Fig. 3). Previous studies reported that 4-HPR monotherapy in ESFT induced apoptosis through p38 MAPK pathway [13] whereas GST monotherapy also induced apoptosis in SK-N-MC cells [36]. Combination of 4-HPR and safingol in SK-N-MC cells markedly increased Bax but no change in Bcl-2 level during normoxic and hypoxic conditions [39]. On the other hand, GST induced alterations in Bax and Bcl-2 levels in breast cancer [17] and neuroblastoma [20] cells. Recent reports showed that GST switched Bcl-2 from an anti-apoptotic protein into a pro-apoptotic protein in breast cancer cells [40] and increased Bax and decreased Bcl-2 leading to an increase in Bax : Bcl-2 ratio for apoptosis in SK-N-MC cells [36]. An increase in Bax and its translocation to mitochondria could induce mitochondrial release of pro-apoptotic molecules [41]. Our treatment with combination of 4-HPR and GST increased mitochondrial release of Smac/Diablo into the cytosol (Fig. 4A). We also found downregulation of the anti-apoptotic BIRC-2 (cIAP1) and BIRC-3 (cIAP2) proteins in Ewing’s sarcoma SK-N-MC xenografts (Fig. 4A). BIRC proteins are upregulated to cause tumor cell survival and resistance to radiation and chemotherapies [42]. Overexpression of BIRC-2 and BIRC-3 also causes inhibition of caspase activation and lack of apoptosis [43], indicating their ability to act as potential oncogenes. On the contrary, Smac/Diablo acts as an indirect activator of caspases by inhibition of the BIRC proteins [44]. We found that combination of 4-HPR and GST caused mitochondrial release of Smac/Diablo in the cytosol to downregulate BIRC-2 and BIRC-3 and facilitate apoptotic process with activation of caspase-3 in SK-N-MC xenografts (Fig. 4A).

Mitochondria also induce caspase-independent pathway of apoptosis by releasing AIF [45]. We explored involvement of caspase-independent pathway of apoptosis in SK-N-MC xenografts by determining the cytosolic levels of AIF. Treatment of xenografts with combination of 4-HPR and GST increased cytosolic levels of AIF (Fig. 4A), suggesting that AIF translocation to nucleus could cause caspase-independent nuclear DNA fragmentation for apoptosis. The anti-proliferative and anti-angiogenic potential of the combination of 4-HPR and GST in RD-ES xenografts were due to inhibition of the survival factor NF-κB and the angiogenic factors VEGF and FGF2 (Fig. 4B). These data agreed with previous reports where 4-HPR [21, 22] and GST [23, 24] produced similar effects in a variety of cancers. Thus, efficacy of combination of 4-HPR and GST also caused downregulation of the survival and the angiogenic factors to facilitate apoptosis with increasing caspase-3 activation (Fig. 4B) and activity (Fig. 4C) in Ewing’s sarcoma RD-ES xenografts.

Our results indicated significant overexpression of both calpain and caspase-12 in course of apoptotic death in Ewing’s sarcoma SK-N-MC xenografts (Fig. 5). Calpain is known to be involved in mitochondrial release of AIF [46] and endoplasmic reticulum (ER) stress-mediated activation of caspase-12 for apoptosis [19]. Active caspase-12 directly promotes caspase-9 and caspase-3 activation for apoptosis [47]. We found that significant upregulation of caspase-3 was associated with apoptotic DNA fragmentation in SK-N-MC xenografts following treatment with combination of 4-HPR and GST (Fig. 6). Also, significant overexpression of AIF in TUNEL-positive cells (Fig. 6) strongly implicated involvement of AIF in apoptosis. 4-HPR causes elevation of Bax to induce mitochondrial release of cytochrome c for activation of caspase-3 and caspase-7 and also to release AIF into the cytosol and its nuclear translocation for apoptosis [48]. In another study, GST increased intracellular free [Ca^2+^] due to its release from ER Ca^2+^ storage, causing activation of the Ca^2+^-dependent calpain and thereby caspase-12 for apoptosis in breast cancer cells [19]. We recently reported that GST increased intracellular free [Ca^2+^] to cause activation of calpain, caspase-12, and caspase-3 in neuroblastoma SH-SY5Y cells for apoptosis [20].

In conclusion, our studies showed that combination of 4-HPR and GST effectively induced multiple molecular mechanisms for increasing apoptosis in Ewing’s sarcoma SK-N-MC and RD-ES xenografts in nude mice. The therapeutic efficacy of combination of 4-HPR and GST in two pre-clinical models of Ewing’s sarcoma needs to be further evaluated in clinical trials in the near future.
